# Coley’s Toxin to First Approved Therapeutic Vaccine—A Brief Historical Account in the Progression of Immunobiology-Based Cancer Treatment

**DOI:** 10.3390/biomedicines12122746

**Published:** 2024-11-30

**Authors:** K. Devaraja, Manisha Singh, Krishna Sharan, Sadhna Aggarwal

**Affiliations:** 1 Department of Head and Neck Surgery, Kasturba Medical College, Manipal, Manipal Academy of Higher Education, Manipal 576104, India; 2 Department of GI Medical Oncology, The University of Texas MD Anderson Cancer Center, Houston, TX 77030, USA; msingh4@mdanderson.org; 3 Department of Radiation Oncology, K S Hegde Medical College, Nitte University, Mangalore 574110, India; tk.sharan@gmail.com; 4 Department of Radiation Oncology, The University of Texas MD Anderson Cancer Center, Houston, TX 77030, USA

**Keywords:** Coley’s toxin, T lymphocytes, dendritic cells, immunosurveillance, immunoediting, tumor microenvironment, head and neck neoplasm, immunotherapy, therapeutic vaccines, checkpoint inhibitors, tumor antigen, monoclonal antibody, natural killer cells, cytolytic T lymphocytes, immunobiology

## Abstract

Cancer immunobiology is one of the hot topics of discussion amongst researchers today, and immunotherapeutic modalities are among the selected few emerging approaches to cancer treatment that have exhibited a promising outlook. However, immunotherapy is not a new kid on the block; it has been around for centuries. The origin of cancer immunotherapy in modern medicine can be traced back to the initial reports of spontaneous regression of malignant tumors in some patients following an acute febrile infection, at the turn of the twentieth century. This review briefly revisits the historical accounts of immunotherapy, highlighting some of the significant developments in the field of cancer immunobiology, that have been instrumental in bringing back the immunotherapeutic approaches to the forefront of cancer research. Some of the topics covered are: Coley’s toxin—the first immunotherapeutic; the genesis of the theory of immune surveillance; the discovery of T lymphocytes and dendritic cells and their roles; the role of tumor antigens; relevance of tumor microenvironment; the anti-tumor (therapeutic) ability of Bacillus Calmette– Guérin; Melacine—the first therapeutic vaccine engineered; theories of immunoediting and immunophenotyping of cancer; and Provenge—the first FDA-approved therapeutic vaccine. In this review, head and neck cancer has been taken as the reference tumor for narrating the progression of cancer immunobiology, particularly for highlighting the advent of immunotherapeutic agents.

## 1. Introduction

Cancer immunobiology is one of the hot topics of discussion amongst researchers today, and immunotherapeutic modalities are among the selected few emerging management approaches to have exhibited a promising outlook [1,2]. Some of the newer Immunotherapeutics, such as pembrolizumab and nivolumab, have already received approval from the Food and Drug Administration (FDA) for their clinical use in head and neck cancer (HNC), and more than 40 candidate vaccines are currently under scrutiny for their potential clinical utility [3,4]. However, although immunotherapeutic approaches have beceome a major focus of cancer research in the recent times, the foundations of immunotherapy had already been laid down more than a century ago, and the current resurgence is aided by the remarkable progress in the field of biotechnology over the last few decades [5,6].

In the modern medical literature, the supposed origin of cancer immunotherapy can be traced back to a few centuries back, wherein a few sporadic published accounts discuss the spontaneous regression of malignant tumors following acute febrile infection [6,7]. Although such reports of infection-induced-remission of cancers existed for centuries, it was the seminal work of an American surgeon, William Bradley Coley, just at the turn of the twentieth century, that eventually paved the way to a then-novel approach of treating the tumors non-surgically, with the aid of an activated immune system [5,6]. Coley’s hypothesis, “an acute infection can induce an anti-tumor immune response and could even eradicate a proliferative disease”, stands valid till today, and this explains a rare and sporadic phenomenon of the spontaneous regression of a diagnosed neoplasm [8]. However, back then, Coley’s hypothesis did not receive substantial attention from the scientific community, primarily owing to the lack of comprehension of the underlying mechanism of this “infection-induced anti-tumor immunity”. Subsequently, greatly benefitted by the pieces of evidence which emerged over the next few decades, the renowned scientists of the 1950s, Frank Macfarlane Burnet and Lewis Thomas, described the theory of immune surveillance, which not only validated Coley’s postulation about the immune activation, but also rejuvenated the field of immunotherapy [9,10]. Since then, immunotherapy has come a long way from being a speculative and inconsequential approach to treat a selected few types of cancers to becoming one of the most promising and reliable therapeutic modalities for several cancers. The eventual realization of Coley’s remarkable contribution to cancer immunobiology has earned him the title of ”Father of cancer immunotherapy” [11]. This review revisits some of the major developments in the field of cancer immunobiology since Coley’s description and discusses the implications of those milestones which have been instrumental in bringing back the immunotherapeutic approaches to the forefront of cancer research.

## 2. Early Developments in Application of Immunology in Cancer Treatment at the Turn of the Twentieth century

### 2.1. First Reports of Infection-Induced Cancer Remission

Although Coley described and propagated the idea of treating malignant disease by inducing a bacterial infection, the credit for the first experimental induction of infection to treat a tumor in the era of modern medicine goes to a German physician, Wilhelm Busch [6,12]. As per a report published in 1868, Busch was fascinated by incidents of spontaneous resolution of sarcoma in some of the patients who had a severe form of bacterial skin infection called erysipelas, and himself induced a severe form of erysipelas in a 19-year-female patient with sarcoma of the neck [12]. Despite the considerable tumor shrinkage within two weeks of infection in Busch’s patient, severe systemic issues prompted aggressive measures to control infection, which in turn led to the relapse of the neoplastic disease. Subsequently, a few other German clinicians too reported the remission of malignant tumors in a few of their patients after induced erysipelas, which they did by injecting the patients with cultured *Streptococcus erysipelas* [6,13,14]. However, these random reports largely went unnoticed, owing to the lack of scientific explanation of the underlying mechanism of this infection-induced anti-tumor activity.

### 2.2. Coley’s Toxin—The First Immunotherapeutic

In 1890, Coley operated upon a 17-year-old female patient with sarcoma, who succumbed to the aggressive disease despite a radical treatment [5,11]. Following the loss of a young patient early in his surgical career, Coley pledged his time and dedication to finding an effective treatment for such an aggressive malignant disease [5,11]. He reviewed numerous case studies and hospital data of his senior colleagues, during which he found an intriguing case of inoperable recurrent sarcoma that had vanished following erysipelas, and on tracing the patient himself, he learned about the patient’s disease-free status seven years following surgery [5,11]. Influenced by the positive response of this case and of similar reports from the past, in 1891, Coley injected a broth culture of *Streptococcus erysipelas* into one of his other patients, a 35-year-old male, who had recurrent spindle cell sarcoma of tonsil and neck [5,11,15,16]. He noted a complete regression of the tumor within two weeks of developing severe erysipelas [15]. The patient reportedly did well for eight years until the fatal relapse of the disease [15]. Coley attributed the therapeutic response seen in this case, and in previous reports, to the reactivation of the immune response [15,16]. In the next few years, Coley published favorable results with this approach in several other patients of sarcoma and carcinoma; however, as noted by Coley himself, good therapeutic responses were apparent only in cases where a frank bacterial infection could be induced by his toxin [15]. Interestingly, a few other immunologist of that era also reported similar outcomes in their patients [17].

By 1910, Coley had used this strategy successfully in numerous sarcoma cases, by injecting mixed toxins of *Streptococcus erysipelas* (now known as *Streptococcus pyogenes*) and *Bacillus prodigiosus* (now known as *Serratia marcescens)*, both as a primary therapeutic as well as an adjuvant after the surgery to prevent recurrences [15]. Despite the breakthrough oncological outcomes of mixed toxins, not many physicians and pathologists of that era accepted Coley’s postulations, and a few even criticized his methods [18,19,20,21,22]. Also, Coley himself had recognized that the infection–remission saga works best in sarcoma; however, he was not able to explain either the mechanism behind the selective activity against sarcoma as well as the lack of predictability of therapeutic responses even amongst sarcoma patients [6]. These drawbacks and the subsequent advent of radiotherapy techniques and chemotherapy drugs overshadowed the therapeutic efficacy of infection-induced anti-tumor immune until the middle of the twentieth century [11]. Nevertheless, Coley continued to use the so-called “Coley’s toxin”, a combination of heat-killed *Streptococcus pyogenes* and *Serratia marcescens*, to cure hundreds of patients with bone and soft-tissue sarcoma over the years [11,23].

### 2.3. Discovery of the “Magic Bullet”

Robert Koch, a German physician who is credited with discovering bacteria causing tuberculosis and cholera, and his proteges, Emil von Behring, Paul Ehrlich, and Friedrich Loeffler, all Germans, and Shibasaburo Kitasato, a microbiologist from Japan, were the prominent personalities belonging to “the golden age of bacteriology” [24]. Their work in the last quarter of nineteenth century not only enabled the identification of causative agents of several bacteria, but also led to discovery of the “magic bullet”, which could impart protection against toxins of specific bacterial infections. In 1884, Friedrich Loeffler acknowledged the etiologic agent of diphtheria as bacteria, as described by Edwin Klebs [25,26]. Klebs had already realized the role of “a circulating toxin in blood stream” as the reason for manifestations of the disease, and Loeffler suggested that neutralizing this toxin could be effective in curing the disease. In 1888, Emile Roux and Alexandre Yersin from the Pasteur Institute, Paris, France, isolated this “circulating toxin” [26]. The same year, Behring also published his report on anthrax and noted that the serum of the rats that were able to kill anthrax bacteria was responsible for the immunity exhibited by rats against anthrax [26,27]. Subsequently, in another experiment, he injected untreated guinea pigs with diphtheria toxin and treated them afterward with sera of animals that had survived the disease, thereby leading to the discovery of the antibodies and the principle of ‘the antitoxin effect of serum therapy’ [26]. Kitasato, in a separate report, also noted similar findings with mice and rabbits immunized with cell-free fluid against tetanus [26]. In 1890, Behring and Kitasato published together on their success in being able to cure animals infected with both diphtheria and tetanus and on how they could protect uninfected animals through passive immunization with antibodies against these bacterial toxins [26,28]. Further, Behring teamed up with Ehrlich, and produced a high-quality, standardized anti-diphtheria serum from dairy cattle, which they distributed to pediatric clinics across Berlin [29]. This serum therapy was hugely successful and reduced the mortality rate of diphtheria drastically between 1892 and 1894 and was hailed unanimously as the best method of cure ever devised till then in pre-antibiotic era [26]. Interestingly, Ehrlich had already noted that exposure to a toxin in small incremental quantities could render the animals immune to subsequent lethal doses, which forms the basis for modern vaccination [29,30]. Behring was awarded a Nobel prize in 1901, the first for medicine and physiology, for his work on serum therapy [26]. Later, Ehrlich also received the Nobel prize in 1908, together with Elie Metchnikoff, for his work in immunology [29]. Nevertheless, the discovery of antibodies, which Ehrlich referred to as the “magic bullet”, has also been a historical milestone in tumor immunobiology, as this understanding laid the foundation for several concepts of immune-mediated tumor cytolysis.

### 2.4. Other Attempts to Induce Anti-Tumor Immunity

Those days many other researchers had also made attempts to cure neoplastic disease by injecting autologous tumor cells into the patients [31]. E. von Leyden and F. Blumenthal were the first ones to attempt this in 1902, by using an autologous tumor cell suspension as the vaccine to treat patients suffering from advanced metastatic disease [31,32]. Although initial attempts were barely successful, some of the subsequent studies reported a measurable clinical response with preparations of autologous tumor cells [31,32,33,34]. These reports account for the first practical attempts at developing therapeutic vaccines to cure cancer [31,35]. Interestingly, the turn of the twentieth century had also seen few reports of tumor remission with viral infection or immunization against one. In 1904, George Dock of Michigan University reported a case of transient remission of acute leukemia after an infection with influenza, suggesting the role of viruses in anti-tumor treatment [36,37]. Although many subsequent studies have reported the spontaneous clinical remissions of neoplastic diseases after natural viral infections such as measles or after immunization with rabies vaccines, Dock’s report was probably one of the first to have discussed virus-induced anti-tumor activity [37]. Over the next few years, several other scientists of that era consistently hypothesized the presence of immune mechanisms responsible for the rejection of transplanted tumors in mice and provided a genetic rationale for the same [7,38,39]. Shortly thereafter, in 1915, James B. Murphy and John J. Morton from the Rockefeller Institute of Medical Research, a dedicated research center in New York, identified the crucial role of circulating lymphocytes in both natural and induced immunity against transplanted tumors in mice [40]. They demonstrated that the experimental prevention of lymphoid reaction failed to offer protection against transplanted tumors [40]. Despite these compelling works, research on tumor immunology did not progress much in the early part of the twentieth century, as not many published studies provided any credible evidence on the immunobiology of cancer.

## 3. Institutional Support and Theoretical Advancements in Cancer Immunology During the Second Half of the Twentieth Century

### 3.1. Establishment of a Dedicated Institute for Research on Cancer Immunology

In 1916, John D. Rockefeller Jr. donated land in New York for the relocation of the only dedicated cancer hospital then, which eventually paved the way for the genesis of the now-iconic Memorial Sloan Kettering Cancer Center (MSKCC) in 1960 [5]. After the demise of Coley in 1936, his daughter Helen Coley Nauts tried reviving interest in Coley’s toxin by reviewing the results of 800+ proven cases [5]. With a grant of USD 2000 from Nelson Rockefeller, son of Rockefeller Jr., and the support of her friend, Oliver R. Grace, she founded the Cancer Research Institute (CRI) in New York in the year 1953 [5,41]. The CRI, by continuing the research on Coley’s postulations, and by supporting works of numerous stalwarts, such as Lloyd J. Old, Rolf Kiessling, Ralph Steinman, Susumu Tonegawa, and many more (whose works are discussed below), has been instrumental in several discoveries that have changed the landscape of cancer immunobiology. Over the years, the CRI has evolved to become a global leader in research on cancer immunobiology and immunotherapy, and has contributed to the development and launch of therapeutics, such as Gardasil, the first preventive human papilloma virus (HPV) vaccine, and more [41].

### 3.2. Genesis of the Theory of Immune Surveillance

In 1958, Tilden C. Everson and Warren H. Cole from Chicago reported a series of 176 cases with spontaneous regression of malignant tumor, including a few metastatic foci [42]. Although these authors could not find any scientific explanation for spontaneous regression, they identified and implicated febrile infection (suggesting activation of the immune response) as a possible reason in several cases. Meanwhile, animal experiments continued to appraise the numerous plausible interactions between tumor cells and the immune system [9]. The following year, Lloyd J. Old and his co-workers from MSKCC, New York, successfully demonstrated the anti-tumor ability of Bacillus Calmette–Guérin (BCG), a vaccine used primarily against tuberculosis, in a mouse model [43]. It was around the same time that Burnet and Thomas proposed one of the much-deliberated concept of that era, the ‘theory of immune surveillance,’ which was inspired by the results of their work on immunological tolerance and also drew support from a few other studies [9,10]. Immune surveillance is the mechanism by which the host immune system can identify the new antigens associated with precursors of cancer and, in most cases, destroy them even before they become clinically apparent. They equated this mechanism to provoking immunological tolerance in homograft failure [9,10]. Burnet received the Nobel prize in 1960 for the discovery of acquired immunological tolerance, which he shared with Peter Medawar, who has contributed immensely to the science of transplantation biology [44,45]. Nevertheless, by virtue of these critical breakthroughs in the field of immunology witnessed in the middle part of the twentieth century, the scientific community could finally comprehend the complexity of immune mechanisms that underlie the anti-tumor activity of infection- or febrile illness-induced immune activation [18,19,20,21,22,46].

## 4. Evolution of Cellular Immunology in Cancer Research

### 4.1. Discovery of T Lymphocytes—The Dawn of the Era of Immunotherapy

Although Murphy and Morton, and a few other researchers before that, had identified the anti-tumor activity of circulating lymphocytes by 1915, not many researchers were able to provide evidence to support the underlying mechanisms until Jacques Miller. Between 1959 and 1962, Miller published the results of a series of his experiments that he had conducted as part of his Ph.D. research topic—‘pathogenesis of mouse leukemia induced by the Gross virus’ [18,47,48,49,50]. By studying the effects of thymectomy in neonatal mice on various situations, such as virus inoculation, thymus tissue grafting, skin grafting, etc., Miller discovered the novel thymic lymphocytes (now called T lymphocytes or T cells) and found evidence on their active involvement in cell-mediated immunity against viruses as well as in countering the immune tolerance proposed by Burnet and Medawar [18,49,50]. Miller, who had been inspired by renowned immunologists like Burnet and Medawar, and Jim Gowans, another prominent immunologist of those days, was lauded by the same stalwarts as his commendable discovery helped them establish the evidence supporting their respective hypotheses on the immune mechanisms. Arguably, this was the most crucial milestone in the evolution of immunobiology that is said to have changed the face and pace of immunological research and its clinical implications forever [20,46,49]. As far as cancer immunobiology is concerned, Miller and his colleagues identified the critical role of T lymphocytes in carcinogenesis and its progression, thus supporting the hypothesis of immunological surveillance of Burnet and Thomas [47,48]. The discovery of T lymphocytes and the realization of the role of cytolytic T lymphocytes (CTLs) in the systemic immune response not only set the wheels of immunology (and immunotherapy) in motion but also opened the floodgates for immunologists across the world to expand the horizons of immunology, including that of cancer immunobiology.

### 4.2. Discovery of Other Key Apparatus of Antigen-Mediated Immune Mechanisms

In 1970, J. C. Cerottini, A. A. Nordin, and K. T. Brunner demonstrated that the action of CTLs can be made specific to a particular antigen-bearing cell by pre-sensitizing them with that target antigen [51,52]. In 1973, Ralph M. Steinman and Zanvil A. Cohn discovered the dendritic cell (DC) in peripheral lymphoid organs of mice, which turned out to be one of the most potent antigen-presenting cells (APCs) [53,54,55]. Further, Rolf Zinkernagel and Peter Doherty published their reports in 1974, in which they proposed the major histocompatibility complex (MHC)-I-restricted CTL recognition mechanism to explain the antigen-specificity of cell-mediated immunity [56,57]. This crucial discovery by Zinkernagel and Doherty got them a Nobel prize in Physiology or Medicine in 1996 [58,59]. Similarly, Steinman’s description of the morphology and tissue distribution of the DC, and his discovery of its crucial role in adaptive immunity, also won him the Nobel prize in Physiology or Medicine in 2011 [53,60]. Interestingly, Steinman shared the Nobel prize with Bruce A. Beutler and Jules A. Hoffmann, who were awarded for their work on Toll-like receptors (TLRs), one of the critical components of the innate immune system responsible for recognizing pathogens [60]. Nevertheless, since these discoveries of the 1970s, the ability to induce an antigen-specific action of CTLs has come a long way to become one of the commonly used strategies of therapeutic vaccines, which generally target a specific antigen expressed on tumor cells to execute an effective as well as selective anti-tumor immunity [4].

### 4.3. Introduction of Alternate Apporaches in Cancer Immunotherapeuctics

Monoclonal antibodies (MABs) are monovalent antibodies which bind to the specific epitope of a cell to execute any given action and are produced from a single clone of Bursa (B)-lymphocyte or B-cells. These MABs have multitudes of anti-tumor mechanisms, from the receptor-based direct killing of tumor cells to the immune-mediated killing and can also act by vascular ablation in stroma [61,62]. The first MAB was produced in 1973, as a hybrid of human and mouse somatic cells, secreting immunoglobulin of both parental types [63]. However, it was the work by Georges Köhler, a German post-doctoral fellow under the guidance of an Argentinian biochemist, César Milstein, that eventually led to a new dimension in the immunobiology of cancer [64,65]. In their landmark paper published in 1975, they demonstrated the ability of hybridoma cells (produced by fusing an antibody-producing plasma cell with a myeloma cell) to propagate indefinitely in culture, enabling the production of unlimited amounts of antibodies in vitro [64,65,66]. Recognizing the impact of their work on immunobiology, Köhler and Milstein were awarded the Nobel prize in Medicine in the year 1984 [65,67]. Since the introduction of the first fully licensed MAB in 1986, MABs have become the fastest-growing group of pharmaceutical molecules, with several refinements of the techniques used for producing MABs [65,66,67]. Interestingly, the mid-1970s saw another crucial discovery that is also emerging as one of the promising immunotherapeutic approaches in the recent times. By 1974, several reports had emerged on ”spontaneous cytotoxicity by the non-thymus-derived lymphocytes”, without the need for matching the MHC antigen of the effectors and tumor cells, as observed in mice, rats, and humans that had no prior exposure to the tumors [68,69]. Subsequently, Rolf Kiessling and colleagues, in their landmark papers of 1975 and 1976, termed those cells as “natural killer” (NK) cells, and they also demonstrated that the ability of NK cells to spontaneously kill a tumor target could also work in vivo [70,71,72]. Further discussion of MABs and NK cells can be found in the last section of this review.

## 5. Therapeutic Vaccines as Prototype of Immunotherapeutic Approaches

### 5.1. BCG—The First Therapeutic Vaccine with Definitive Indication

BCG, an attenuated strain of *Mycobacterium bovis*, was isolated in 1908 by Albert Calmette and Camille Guérin from glycerol bile potato medium at the Pasteur Institute in Lille, France, which was followed by its first human use in 1921, as a vaccination against tuberculosis [73]. A few years after this, Raymond Pearl of Johns Hopkins University suggested a potential antagonism between cancer and tuberculosis, as he observed active tubercular lesions in 6.6% of those with malignant lesions as compared to 16.3% in those without malignancy, in an autopsy study with a sample size of 816 [74]. Nevertheless, attention towards the utility of BCG as an anti-tumor agent got intensified only after the first successful attempts to induce anti-tumor immunity with BCG in a mouse model by L. J. Old and his colleagues in 1959 [43]. By the 1970s, several studies, both animal and clinical, documented the activation of the immune system with the BCG vaccine, which paralleled its anti-tumor abilities in the form of measurable regression of several types of solid tumors such as superficial cancer of the bladder, hepatocellular carcinoma, adenocarcinoma of the kidney, malignant melanoma, and HNC [75,76,77,78,79]. Particularly, the clinical work of two urologists from Canada, Alvaro Morales and David Eidinger, in patients with kidney and bladder cancers provided the necessary momentum for the advancement of immunotherapeutic research with BCG [75,76]. Meanwhile, in 1975, L. J. Old and colleagues from MSKCC, New York, identified an endotoxin-like substance called tumor necrosis factor (TNF), which was responsible for necrosis of transplanted tumors in mice [80]. TNF later turned out to be one of the crucial discoveries of Old, as a key cytokine involved in cytolysis of cancer cells. L. J. Old’s contributions via his mouse studies with BCG, and the discovery of TNF, have earned him the shared title of ‘father of immunotherapy,’ with Coley [7,81]. Nevertheless, encouraged by these results and the then newly-discovered concept of sensitizing CTLs, some of the investigators of that era mixed irradiated autologous tumor cells with the BCG, in an attempt to enhance and direct the resultant immune response towards the tumor cells, and were able to elicit a desired immunological as well as clinical activity [82,83,84,85]. Although BCG monotherapy, by then, was known for its potent immune stimulation mechanisms locally (via direct suppression of tumor growth as well as by effective recruitment of immune cells and through increased expression of interferon-gamma (IFN-γ)), it is the role of BCG as an adjuvant to other active agents that caught the attention of the researchers of that time, probably for the increased anti-tumor ability as a combination therapy compared to the monotherapy [86,87,88]. Nevertheless, BCG monotherapy, supposedly the first therapeutic vaccine to find a definitive clinical application and with predictable outcomes, continues to be a clinically reliable topical agent for treating superficial bladder cancer even after 50 years of its introduction [86,87,88].

### 5.2. Central Mechanism of Therapeutic Vaccines

These reports on the anti-tumor ability of BCG marked the beginning of a systematic transition of immunotherapeutic approaches from being ‘an inducer of non-specific immune response that could kill tumor cells’ towards becoming a ‘specific immune mechanism that exhibits cell-mediated cytotoxicity against tumor cells by producing antigen-specific CTLs.’ The researchers of those days used the term active specific immunotherapy (ASI) to define the specific anti-tumor immunological response observed in their studies after the injection of an active agent (inactivated tumor cells) and an adjuvant (BCG) [82,83,84,85]. ASI, also known as antigen-specific immune response (ASIR), tumor-specific immune response, or specific immune-mediated tumor rejection, can be defined as a process in which an exogenous agent is administered to induce an active cell-mediated immunity against the specific antigen expressed by a particular tumor cell [82,83,84]. Most of these earlier studies used BCG cell wall or Newcastle disease virus-based vaccines to deliver either live or irradiated autologous tumor cells to induce ASI [82,83,84]. In one of the studies, although the vaccine containing living tumor cells cured most of the guinea pigs, it induced tumors at the vaccine sites in a few animals. Contrastingly, the vaccines with irradiated tumor cells were not tumorigenic but required more tumor cells for successful therapy [82]. Nevertheless, by this time, it had become apparent that the APC recognized the specific antigens expressed on tumor cells and presented them to the immune system, eventually activating appropriate T cell responses. It had also been realized by then that the production and release of the antigen- (tumor-)specific CTL is essential for anti-tumor immunity, as this antigen-specific cell-mediated cytotoxicity is the central process involved in the elimination of neoplastic cells [89,90]. It is interesting to note that the authors of that era had appreciated the role of innumerable antigens (then unknown) that could be activated by a bacterial infection or a traumatic event and, in turn, stimulate the host immune response, which at times, even resulted in spontaneous regression of an invasive tumor [91]. Despite these glaring pieces of evidence and sound hypotheses, there were a few missing links, particularly regarding how invasive tumors could evolve despite immunosurveillance. These critical questions, along with some of the contradicting reports of therapeutic responsiveness to various immunotherapeutic approaches and the technical hurdles of those days, all culminated in doubts about veracity of theory of immunosurveillance [92,93,94]. On the other hand, those promising responses of the earlier works with BCG encouraged the scientists to look for safer and more effective immunotherapeutic agents. Several key developments at the turn of the twenty-first century provided the needed impetus in this regard.

### 5.3. Melacine—The First Therapeutic Vaccine to Be Engineered

In a phase I trial, Mitchell et al. from the University of Southern California School of Medicine, Los Angeles, injected the mechanical lysates (homogenates) of two allogeneic melanoma cell lines with a novel adjuvant called DETOX (detoxified Freund’s adjuvant, Ribi ImmunoChem Research, Inc., Hamilton, MT, USA) in 22 patients of melanoma, and found complete remission of the disease in two patients, partial response in three patients, and minor responses in another three patients [95]. In their results, published in 1988, they noted elevated levels of CTL precursors in all eight patients who had a clinical response, and none of the seven patients with no increase in their CTL precursors had any clinical response. Subsequently, the authors repeated the same experiment in another set of 25 patients with metastatic melanoma and found that the ASIR induced by these allogeneic lysates of melanoma with adjuvant DETOX could induce regression of the disease in a proportion of patients and with little toxicity [96]. Here also, an increase in CTL precursors correlated well with the clinical outcome. These results led to the engineering of the first therapeutic vaccine in 1998, called Melacine (Ribi ImmunoChem Research, Hamilton, MT), developed as a lyophilized preparation from the same two allogeneic melanoma cell lysates used by Mitchell et al. [97]. The trials from other centers too reported tumor regression with Melacine in around 10% of the patients, with long-term stabilization of disease in 10% to 20% [97]. Notably, in these studies, the benefit of Melacine was best appreciated in an adjuvant-treatment setting [98]. Interestingly, at around the same time, researchers from John Wayne Center Institute, Santa Monica, CA, also reported promising results in advanced cases of melanoma, with another therapeutic vaccine called Canvaxin, which was an autologous whole tumor cell-based polyvalent vaccine admixed with BCG [85]. However, subsequent phase III randomized controlled trials on Melacine and Canvaxin failed to demonstrate any advantage of these vaccines in the adjuvant-treatment settings of melanoma patients [99,100]. Also, the adjuvants used in these vaccines, DEXOT and BCG, though immunogenic, had a few safety concerns [101,102]. Nevertheless, since the introduction of Melacine and Canvaxin, our understanding of tumor immunobiology has progressed in leaps and bounds, and consequently, its applications, including the development of therapeutic vaccines, have also come a long way. Interestingly, among the several transforming changes or inventions seen over the years, the three major developments that can be credited for the metamorphosis of therapeutic vaccines are all from around the same time as the design and evaluation of Melacine.

### 5.4. Enhanced Ability to Measure the Elicited ASIR

In 1983, Cecil C. Czerkinsky and colleagues from Sweden introduced a new high-throughput technology called enzyme-linked immunosorbent spot (ELISpot) assay, a versatile and sensitive alternative to the conventional plaque-forming cell assays that were used to enumerate cells involved in immune response till then [103]. The high accuracy, sensitivity, reproducibility, and robustness of ELISpot led to its subsequent widespread use for measuring cell-mediated ASIR in both mice and humans, quantitatively. Owing to its ability to measure a broad range of magnitudes of response, including the critical ASIR activities (for measuring cytotoxicity) such as IFN-γ secretion and granzyme B release, CTL monitoring by ELISpot is one of the current gold-standard methods for the evaluation of immune responses in clinical trials and vaccine candidature studies [104,105].

### 5.5. Isolation of DCs

DCs, dubbed nature’s adjuvant, are known for their exceptional capacity to initiate both innate as well as adaptive immune responses [106]. The isolation of DCs from bone marrow precursors in the 1990s eventually led to the development of DC-based therapeutic vaccines that quickly became the preferred vehicles and adjuvant for the development of vaccines [107,108]. As DCs are critically involved in (tumor-) antigen presentation to the host immune system, conjugating DCs with a tumor antigen potentiates the induced ASIR against the tumor cells. The realization of the role played by the adjuvant in increasing the therapeutic response of a vaccine has led to the discovery of several other potent adjuvants in recent times, such as Montanide ISA, based on detoxified or Freund’s incomplete adjuvant (IFA), TLR-based adjuvants, granulocyte–macrophage colony-stimulating factor (GM-CSF), polylactic acid, polylactide-coglycolide, and imiquimod [4,101,102].

### 5.6. MAGE—The First Tumor Antigen to Be Recognized

Tumor antigens are nothing but antigens expressed by tumor cells that can be exploited as specific targets for inducing ASIR. Around the same time as the development of Melacine by Mitchell et al., several other investigators identified a few novel tumor antigens and documented their efficiency in inducing ASIR as well as their ability to hinder the growth of tumors in experimental models [89,109,110]. A tumor antigen identified by Pierre van der Bruggen et al. in 1991, called melanoma antigen-encoding gene (MAGE), encoding a tumor antigen of melanoma, was the first real molecular target identified for the induction of ASIR [110]. Since then, batteries of new and potent tumor antigens have been recognized in various tumors, paving the way for development of therapeutic vaccines belonging to different classes [111,112]. As for HNC, the envelope proteins E6 and E7 of HPV 16 and 18 are the most attractive and widely targeted tumor antigens for developing therapeutic vaccines [4]. The involvement of these oncogenic viruses in the carcinogenesis of HPV-driven HNC offers a peculiar advantage by providing an opportunity to selectively target those HPV antigens harboring tumor cells and sparing the normal cells [4,113]. Tumor antigens can be broadly classified as shared antigens, expressed in tumor cells as well as in normal tissues, and tumor-specific antigens, expressed only in tumor cells. Alternatively, they could be classified based on their peculiar expression characteristics as cancer-testis antigens, oncoviral antigens, overexpressed/differentiation antigens, neoantigens, and stealth antigens [114,115,116,117]. In 2009, the Translational Research Working Group of the National Cancer Institute (NCI) published a report on its pilot project, whose objective was to prioritize and rank the tumor antigens based on their immunogenicity (17%), oncogenicity (15%), specificity (15%), and five other criteria [118]. Using the analytic hierarchy process, the expert committee of NCI found that none of the 75 evaluated antigens could satisfy the requirements to be called an “ideal” tumor antigen [118].

### 5.7. Provenge—The First FDA-Approved Therapeutic Vaccine

Sipuleucel-T (Provenge, Dendreon Pharmaceuticals LLC, Saturn Way, Seal Beach California, US), an autologous immunotherapeutic agent designed to stimulate an ASIR against prostate cancer, showed a definitive clinical response and survival benefits among men with metastatic castration-resistant prostate cancer (mCRPC) in early clinical studies [119,120]. Sipuleucel-T contains autologous peripheral-blood mononuclear cells, including APCs, activated ex vivo with a recombinant fusion protein—PA2024. PA2024 consists of prostatic acid phosphatase (PAP), a tissue antigen over-expressed by prostate cancer cells, fused to GM-CSF as an adjuvant [121]. When injected intravenously, sipuleucel-T stimulates an immune response to prostate cancer cells by targeting PAP and generating specific T cells capable of recognizing and killing prostate cancer cells that express PAP [122]. Favorable evidence from early clinical studies led to a double-blind, placebo-controlled, multicenter phase III trial (NCT00065442), called Immunotherapy Prostate Adenocarcinoma Treatment (IMPACT), which was carried out between July 2003 and January 2009, with 512 asymptomatic or minimally symptomatic patients of mCRPC. In this trial, three two-weekly intravenous infusions of sipuleucel-T led to a relative reduction in the risk of death by 22%, representing a 4.1-month advantage in medial survival over the placebo [121]. At three years, sipuleucel-T increased the overall survival by 38% (31.7% in the sipuleucel-T group versus 23.0% in the placebo group) [121]. These results prompted the FDA to approve sipuleucel-T in 2010 for treating patients with asymptomatic or minimally symptomatic mCRPC [122]. Sipuleucel-T is the first and the only FDA-approved therapeutic vaccine for clinical use to date. However, several approaches and agents are currently being evaluated for their safety and efficacy as therapeutic vaccines in different cancer lines, including HNC. A few of these agents, particularly those targeting tumor antigens of HPV-16/18, have already received the Fast Track designation by the FDA for use in recurrent/metastatic/progressive oropharyngeal cancers [4]. Further details on the various carriers, delivery approaches, and candidates of the therapeutic vaccines for HNC can be found in our recent comprehensive review [4]. Some of these agents can potentially change the landscape of cancer chemotherapy for the better.

## 6. Explosion of Immunological Concepts—At the Turn of the Twenty-First Century

### 6.1. Discovery of Immune Tolerance Mechanisms

By 1990, it was discovered that the activation of CTL by tumor antigen-led ASI is also associated with the co-induction of a specific class of immunoglobulins that could counter the activity of CTL itself [123,124]. These controls are primarily meant to serve as immune checkpoints (ICs) to prevent collateral damage due to immune responses against the self-cells, and their activation leads to suppression or induced dysfunction of CTLs, typically in the form of T cell exhaustion, eventually hindering their anti-tumor activity [7]. These findings substantiated the hypothesis of immune tolerance and provided early evidence of mechanisms by which the tumor cells can effectively negate the host immune response. Particularly, of the several identified ICs that attenuate the activity of T cells, the two prominent ones, named cytotoxic T lymphocyte protein 4 (CTLA-4) and programmed cell death protein 1 (PD-1), have attracted a lot of attention, and these ICs shown to be particularly crucial in the conceptualization of immune-tolerance or immune-escape mechanisms [125,126,127]. The impact of the discoveries of PD-1 and CTLA-4 on cancer immunobiology and immunotherapy was so disruptive that Professor Tasuku Honjo and Professor James Allison, who were, respectively, responsible for these discoveries, have been bestowed with multiple laurels, including a Nobel prize in 2018 in Physiology or Medicine [128,129]. Pembrolizumab and nivolumab, both MABs that act by blocking the activity of PD-1, are the only two IC-based immunotherapeutic medications to have received FDA approval for use in HNC to date [3].

### 6.2. Theory of Immunoediting

In the early 2000s, overwhelmed by the growing body of evidence supporting the temporal and spatial dynamicity of the interactions between the immunogenic and the tolerogenic mechanisms, Dunn et al. proposed the peculiar hypothesis of cancer immunoediting, explaining the dual role played by the immune system, via both host-protective mechanisms as well as tumor-sculpting functions, throughout the process of carcinogenesis [130,131]. According to this theory, cancer immunoediting represents a dynamic process encompassing three phases—elimination, equilibrium, and escape. When an oncogenic stimulus induces the process of carcinogenesis, the precursor cells express distinct tumor-specific markers and generate proinflammatory immune responses that take part in the process of immunoediting. In the first phase of the process, called the elimination, which represents the classical concept of cancer immunosurveillance, the cells and the molecules of innate and adaptive immunity work to eradicate a developing tumor. However, if this process is not successful in destroying the tumor cell, the second phase of immunoediting, called the equilibrium phase, sets in, wherein the viable tumor cells are either maintained chronically or immunologically sculpted by immune “editors” to produce new variants of tumor cells selectively. When these newly produced variants eventually evade the immunological restraints of the equilibrium phase, they manifest as clinically detectable tumors in the third and final phase of immunoediting, the escape phase [131]. At the outset, the proposed model of immunoediting and these underlying mechanisms not just answer most of the earlier questions related to immune tolerance but has also found substantial evidential support in the latest studies [131]. Moreover, the acknowledgment of these immunoediting processes, particularly the comprehension of the dynamic changes at the level of the tumor microenvironment (TME), has played a critical role in bringing immunotherapeutics closer to clinical application than ever before [131].

### 6.3. Role of the TME in the Process of Immunoediting

The balance between immune surveillance and immune escape is a result of a complex interplay between numerous cellular proteins and pathways, determined by several genetic, environmental, and tumor-related factors that vary drastically between individuals. The cancer–immune set point is the equilibrium between factors that promote and suppress anticancer immunity, and a delicate balance of this set point is essential for recognizing and eliminating tumor cells at one end and preventing autoimmunity on the other [127]. At the molecular level, induction of CTL-mediated ASI is one of the primary mechanisms of anti-tumor immunity, which is carried out by releasing cytokines such as IFN-γ/TNF-α, perforin, and granzyme that eradicate cancer cells [132,133]. On the other hand, the principal mechanism of hindering anti-tumor immunity includes countering the activation, differentiation, or release of T cells or attenuating their function in the TME, in which ICs play a crucial role. Some of the other mechanisms known to exhibit anti-immunity action include (a) activation of immunosuppressive cells such as tumor-associated macrophages (TAMs), T/B-regulatory cells (T/B-regs), myeloid-derived suppressor cells (MDSCs), (b) secretion of immunosuppressive cytokines, like transforming growth factor-β, (c) activation of immunosuppressive signaling pathways, like signal transducer and stimulator of transcription-3, and (d) formation of physical barriers and intricate vascular networks within the tumor, that hinder drug delivery and render the cells hypoxic [4,134]. Though several of these molecular mechanisms were recognized later, it had already become apparent a few decades ago that immune surveillance represents only one dimension of the intricate interactions between the host immune system and factors responsible for carcinogenesis [130,135]. Cancer immunotherapeutics, including therapeutic vaccines, act either by producing a robust ASIR against the tumor cells or by negating the immunosuppressive conditions/factors to enable the reactivation of naturally existing anti-tumor immunity [4].

### 6.4. Immunophenotyping of Cancer

As per the current understanding of cancer immunology, malignant tumors can be broadly classified into three types of immunophenotypes, depending on the relative abundance and distribution of immune cells in the TME [134]. Tumors with high T cell infiltration, high PD-L1 expression, and exhibiting high tumor mutational load (TMB) are categorized as ‘immunoinflammatory tumors.’ In ‘immune-excluded’ or ‘immune-rejected tumors,’ the cytotoxic T cells are often blocked at the surface without being able to enter cells to exert cytotoxic effects. Lastly, ‘immune-desert tumors’ are characterized by few or no CTLs, low PD ligand-1 (PD-L1) expression, and low TMB [134]. Each of these cancer-immunophenotypes seems to have specific underlying molecular mechanisms of immune escape that prevent the host’s immune response from eradicating cancer [127]. For instance, while the hyper-exhaustion of T cells by the ICs is attributed majorly to immune escape in inflamed tumors, the presence of immunosuppressive cells such as TAMs, T/B-regs, MDSCs, and a hypoxic microenvironment are held responsible for the immunosuppressive TME associated with non-inflamed tumors [127,134]. Interestingly, the profile of recruited immune cells that contribute greatly to the immunophenotype of a particular tumor also dictates its hallmark traits like biological behavior and therapeutic responsiveness [136,137,138]. For instance, the immunoinflammatory types of tumors respond efficiently to immunotherapeutic strategies like inhibition of ICs (with anti-PD-L1/PD-1 therapy), owing to the pre-existing anti-tumor immune response that becomes rejuvenated by these agents. Contrastingly, non-inflamed tumors respond poorly to this approach as they have few or no CTLs and exhibit low PD-L1 expression [127]. In other words, for therapeutic actionability, the tumors with immune-desert and immune-excluded phenotypes are considered ”cold tumors”, as against the ”hot”’ and actionable inflamed tumors [139,140,141].

## 7. Other Prominent Immunotherapeutic Modalities

### 7.1. Adoptive Cell Therapies (ACTs)

In later half of 1980s, Steven Rosenberg, from NCI, in separate experiments with a mouse model and in humans, demonstrated the ability to achieve partial and complete responses in advanced cancers with the administration of high doses of a cytokine, interleukin-2, and CTLs, independently and in combination with each other [142,143]. This was the first report of adoptive transfer therapy (ATT) or ACT that exhibits anti-tumor activity via an expressed T cell receptor (TCR) which recognizes target cells in an MHC-independent manner [4,144,145]. The ACT involves harvesting of CTLs from freshly resected tumor tissues, followed by their expansion and reinfusion into the patients, which not only is resource-consuming, but the response is also not predictable. Although the transfer of CTLs with tumoricidal properties can aid in overcoming the resistance to tumor antigens, its efficacy is determined by the presence of pre-existing tumor-reactive T cells in patients that could be affected by the immunoediting process discussed earlier [145,146]. The TME could also assist in immune escape by aiding the survival of tumor cells against these infiltrated effector cells [145,146]. Nevertheless, advances in biotechnology have helped in overcoming these limitations of conventional ACT by the introduction of engineered T cells that encode an exogenous TCR and the chimeric antigen receptor (CAR), an artificial receptor with an extracellular region that is able to recognize cancer antigens and an intracellular fragment to initiate activation of CTLs [146]. The concept of the chimeric T cell receptor was introduced by Japanese immunologists in 1987, and CAR-T cell therapy, a form of ACT that bypasses the need for tumor cells to possess a functional antigen processing machinery, was introduced by immunologists from Israel in 1989 [144,145,147,148]. Further, backed by the results of subsequent studies, the FDA approved the first CAR-T cell therapy in 2017, for use in hematological malignancy. Subsequently, six more therapies are approved till April 2023, for use in patients with B-cell malignancies and multiple myeloma [144]. While CAR-T therapy has shown promising outlook with predictable results, there are several concerns associated with its clinical use [146]. Apart from high costs of production, storage, and transportation of CAR-T cells, there are a few serious adverse effects that can occur with therapy, in the form of graft-versus-host disease (GvHD), cytokine release syndrome (CRS), and immune effector cell-associated neurotoxicity syndrome (ICANS) [144,145]. Also, with a few exceptions, CAR-T cell therapy has failed to demonstrate reliable anti-tumor activity in solid cancers [144]. Further discussion of the design and development of CAR-T cells can be found in another review article [149]. Nevertheless, with the ongoing efforts to overcome these hurdles, immunologists are hopeful of more effective and safer CAR-T cell therapies in the near future [144]. Moreover, the remarkable achievement of CAR-T cell therapy has led to engineering of other immune cells for immunotherapeutic agents [144,145,150].

### 7.2. MABs

As mentioned in previous sections, MABs are among the fastest growing group of immunotherapeutics, and with the help of latest biotechnological processes, fully humanized MABs have already been made available for the therapy of various types of cancers in humans [65,66,67]. Since the introduction of the first fully licensed MAB in 1986, for use in cases of acute transplant rejection, more than six dozen MABs have received FDA approval for various clinical indications in humans, 30 of which are against cancer [65,66,151]. Rituximab, a chimeric MAB against CD20, was the first anti-tumor MAB to receive approval for use in hematological malignancy in 1997, and trastuzumab was the first humanized MAB to be approved for use in solid cancers in 1998 [151]. To date, four MABs have been approved for use in HNC, as an adjuvant treatment with radiotherapy or chemotherapy or as palliative treatment in recurrent/metastatic tumors [3,151,152]. Apart from pembrolizumab and nivolumab, both humanized MABs against PD-1, cetuximab, a chimeric MAB, and nimotuzumab, a humanized MAB, both targeting a pro-oncogenic protein epidermal growth factor receptor (EGFR), are the approved immunotherapeutics in HNC [3,152]. Numerous tumor antigens, belonging to various categories, have already been identified as targets for the development of MABs that are more effective and well-tolerated [61]. Of several MABs that have been tested, the one against vascular endothelial growth factor, called bevacizumab, and the other inhibitors of ICs, such as ipilimumab and tremelimumab, both against CTLA-4, are among the most-promising molecules to have been introduced into clinical practice [3,61,151]. Further discussion of various MABs in HNC can be found in one of our previous reviews [3].

Other avenues of MABs that are under development are conjugating antibodies with a cytotoxic drug resulting in a superior efficacy and safety, and the development of novel bi- and trispecific antibodies to enhance therapeutic benefit by counteracting each other’s resistance [153]. Two of the MAB–drug conjugates have also obtained FDA approval in the last decade, and another 40 MAB–drug conjugates are under trial in different types of cancers [154]. Interestingly, the combination of an antibody protein that targets a tumor antigen and a toxin protein (in the form of diphtheria toxin), received FDA approval for the treatment of cutaneous T cell lymphoma in 1999 [154,155]. These molecules, called immunotoxins, are made of a recombinant protein consisting of an antibody or antibody fragment targeting a tumor antigen linked to either protein toxins derived from bacteria such as Pseudomonas and Diphtheria toxins, plant toxins such as ricin and gelonin, or some endogenous protein of human origin such as ribonucleases (RNases) and granzymes [155]. Currently, about a dozen immunotoxins are under clinical trials [155].

### 7.3. NK Cell-Based Therapies

NK cells are lymphoid cells of the innate immune system that monitor cell surfaces of autologous cells for an aberrant expression of MHC class I molecules and cell stress markers [156]. The discovery of NK cells explained a previously unknown third mechanism of (T cell-independent) cytotoxicity apart from CTL-mediated cytotoxicity and antibody-dependent cellular cytotoxicity [157]. Nevertheless, since their discovery in 1975, our understanding of NK cells has evolved over the years, and currently, these cells are known as “highly sophisticated innate lymphoid cells that detect harmful changes in cellular self and as pivotal catalyzers of adaptive T-cell responses” [156,158]. In humans, NK cells comprise about 5–20% of peripheral blood lymphocytes, and they can directly eradicate the tumor cells by cytolysis, via granzyme B and perforin, or indirectly contribute by inducing an efficient T cell-mediated anti-tumor response [156]. Due to their intrinsic cell-killing ability, and lack of clonal expansion that can limit their toxicity, NK cells have been viewed as one of the most promising immunotherapeutic agents. Studies have shown that NK cell activity correlates with a reduced risk of cancer [159,160]. As a result, several immunotherapeutic approaches have been explored with NK cells; some of the prominent ones include NK cell-activating therapies, in the form of infusion of cytokines such as interleukins-2 and -15, ACT, by infusing activated autologous or allogenic NK cells, and genetically modified NK cell therapy with the infusion of CAR-NK cells [145,157,161]. Similar to CAR-T cell therapy, in CAR-NK cell therapy, the genetically engineered NK cells encode CARs that recognize tumor antigens [145]. Studies have shown a high response rate in hematological tumors with CAR-NK cell therapy. CAR-NK cell therapy has advantages over CAR-T, in terms of NK cell source, ease of manufacture, and lack of side effects, such as GvHD and CRS [145]. However, these therapies come with several challenges, both technical and practical, and the most important of which is the short half-life of NK cells with resultant reduction in antitumor efficacy [145,161]. Recently, another class of NK cell-based immunotherapeutic modality, called NK cell engagers (NKCEs), has shown promising results by overcoming several of these limitations of NK cell-based therapy, but also of T cell-based immunotherapies, including CAR-T cell therapy [145,161]. NKCEs are synthetic molecules built from fragments of bispecific or trispecific MABs targeting both tumor antigens and NK cell activation receptors, which in turn bring NK cells and tumor cells together and trigger NK cell-mediated killing of tumor cells [145,150]. The trispecific NKCE, which targets two activating receptors of NK cells and one tumor antigen, has been shown to have more potent anti-tumor activity than bispecific agents [161,162]. Recently, several experimental studies have demonstrated the ability to engineer multi-specific NKCEs with enhanced versatility and therapeutic efficacy against different cancer types [163,164]. These NKCEs are easier to manufacture and less expensive than other NK cell-based therapies [161].

### 7.4. Nuclear Factor-κB (NF-κB)

NF-κB, also known as nuclear factor kappa-light-chain-enhancer of activated B cells, was discovered in 1986 by Ranjan Sen, under the guidance of another Nobel laureate (awarded in 1975, for discovering enzyme reverse transcriptase) David Baltimore [165,166]. NF-κB forms a family of transcription factors that mediates numerous cellular pathways, including those responsible for cellular proliferation and apoptosis, and thus it plays an active role in several key oncogenic mechanisms [167,168,169,170,171]. In fact, NF-κB activation has been shown to affect all six hallmarks of cancer through the transcription of genes involved in cell proliferation, angiogenesis, metastasis, inflammation, and suppression of apoptosis [167,172]. In addition, the dysregulation of NF-κB and the related signaling pathways is also shown to play a major role in modulating cancer therapy efficacy, by promoting resistance to chemotherapy as well as radiotherapy [172,173]. As a result, several anti-NF-κB agents are under trial for their therapeutic utility in numerous cancers, including HNC [172]. Aspirin, sodium salicylate, and dexamethasone have shown to suppress NF-κB activation. Similarly, curcumin, also known as diferuloylmethane, derived from plants of the *Curcuma longa* species, and celecoxib, a well-known nonsteroidal anti-inflammatory drug and cyclooxygenase-2 inhibitor, have also exhibited a reliable and selective NF-κB-inhibiting activity against tumor proliferation and progression [174]. Anti-TNF-α MABs, such as infliximab, adalimumab, and anti-golimumab antibody, that have been evaluated for their disease stabilization effect in cancer, also act by hindering NF-κB activation [171]. Combining NF-κB inhibitors with standard cancer therapies, such as chemotherapy, endocrine therapy, and immunotherapy, including IC inhibitors, could help in sensitizing tumor cells to the cytotoxic effects of the drugs, overcoming resistance mechanisms, and potentially augmenting the anti-tumor immune response. These combinations have shown promising results in various cancer types, including cisplatin-resistant HNC [171,173]. Particularly, studies have shown promising results with co-administration of NF-κB inhibitors together with checkpoint blockade [174,175]. One of the major limitations of inhibition of NF-κB or its signaling pathways is that it could also suppress the global immune response and ability to fight bacterial infection, which impedes the long-term use of these inhibitors [171,172,176].

**Figure 1:**
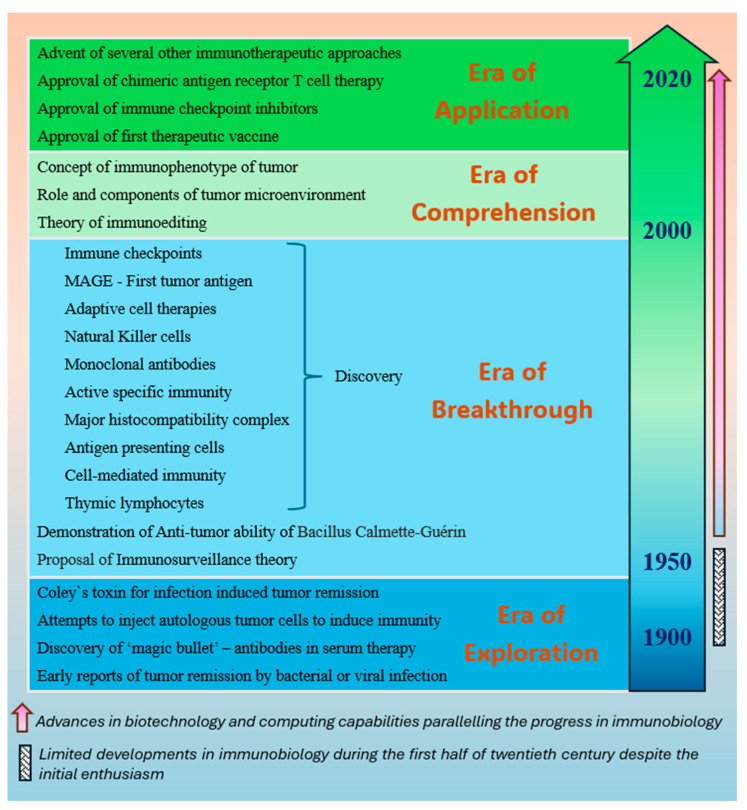
Illustration depicting the evolution of cancer immunobiology leading to introduction of several therapeutic avenues, such as therapeutic vaccines, checkpoint inhibitors, adaptive T cell therapy, monoclonal antibodies, and more.

Figure 1 summarizes the strides made by the immunobiology of cancer that have culminated in expanding clinical applications in recent times, and Table 1 lists some of the key agents that have received approval, are being fast-tracked, or have shown promising outlook in early clinical studies for use in HNC.

## 8. Prospects of Immunotherapy

Immunotherapeutic approaches have become a clinical reality in the last few decades, because of the strong foundation that has been laid by the work of numerous immunologists of the twentieth century along with the rapidly advancing technology of the twenty-first century. Despite all the progress that has been made, there is still a lot to be accomplished with respect to improving the effectiveness and safety of immunotherapeutics, particularly in solid cancers. Apart from making them safer and more tolerable, with fewer or no adverse effects, it is essential to enhance their clinical feasibility and affordability. While IC inhibitors and other CTL-mediated therapies have shown robust clinically activity, they are prone to immune editing mechanisms that limit their therapeutic effect. Newer avenues such as NKCE, CAR-NK cell therapy, co-stimulating strategies, can overcome these limitations of the traditional immunotherapeutic approaches. Particularly, individualizing the choice of immunotherapeutic as per the immunophenotype of the tumor could be one way to enhance effectiveness [180]. Further, approaches to convert the ”cold” and immune depleted tumors to ”hot” and actionable tumors are undergoing development and validation trials [134,141]. On the other hand, to overcome the affordability issues of conventional immunotherapeutics, cheaper alternatives are also being tried as reagents, markers, carriers, and platforms for delivering immune-based therapies [181].

## 9. Conclusions

Though immunobiology-based avenues are re-emerging as potential game changers in cancer therapeutics, the principles behind these approaches are not new. From earlier reports of spontaneous regression of malignant tumors following acute febrile infection to the approval of the first immunotherapeutic by the FDA for clinical use, the approaches and modalities of immunotherapy have come a long way. Particularly, milestones like the discovery of T lymphocytes and DCs, and the anti-tumor (therapeutic) ability of BCG and other vaccines, and the development of theories such as immunosurveillance and immunoediting have all contributed to this extraordinary journey. Further, in-depth comprehension of cancer immunobiology also has led to newer and promising approaches of CAR-T cell therapy and NKCE which have the potential to offer better and safer immunotherapeutics in times to come.

## Figures and Tables

**Table 1:** Summary of the major immunotherapeutic approaches in cancer treatment.

Group	Agent	Mechanism of Action
Therapeutic vaccines	ISA 101 *	Peptide vaccine targeting HPV-16 E6/E7
	CIMAvax	Recombinant human EGF-rp64k
	IPI-549	A specific PI3K inhibitor
	UCPVax	Universal cancer peptides derived from hTERT
	UV1 *	Three synthetic long peptides derived from hTERT
	VTX-2337	Toll-like receptor 8 agonist
	PDS0101 *	Liposomal-based HPV-16 E6/E7 multipeptide vaccine
	CMP-001	Virus-like particle containing Toll-like receptor 9 agonist
	N 803	Interleukin-15 superagonist complex
	NT-I7	Recombinant human interleukin-17
Immune checkpoints	Pembrolizumab **	Anti-programmed cell death 1 antibody
	Nivolumab **	Anti-programmed cell death 1 antibody
	Durvalumab **	Anti-programmed cell death ligand 1 antibody
	Tremelimumab	Anti-cytotoxic T lymphocyte-associated protein 4
	Ipilimumab **	Anti-cytotoxic T lymphocyte-associated protein 4
	Atezolizumab **	Anti-programmed cell death-ligand 1 antibody
	Avelumab **	Anti-programmed cell death-ligand 1 antibody
	Cemiplimab **	Anti-programmed cell death 1 antibody
Monoclonal antibodies	Rituximab **	Anti-CD20
	Cetuximab **	Anti-epidermal growth factor receptor antibody
	Palbociclib	Anti-cyclin-dependent kinase 4/6
	Monalizumab	Anti-NKG2A antibody
	Ramucirumab **	Anti-vascular endothelial growth factor receptor 2
	Bevacizumab **	Anti-vascular endothelial growth factor A
	Panitumumab **	Anti-epidermal growth factor receptor antibody
	Trastuzumab **	Anti-HER 2
	Denosumab **	Anti-RANKL
NK cell-based therapies	NK Cell ADCC	NK cell antibody-dependent cellular cytotoxicity
	CAR-NK cell therapy	Adoptive transfer of genetically engineered CAR-NK cells
	BiKE	Bispecific killer cell engager
	TriKE	Trispecific killer cell engager
Adaptive cell therapy	Infusion of CTL	Adoptive transfer of autologous or allogenic CTLs
	TCR-T cell therapy	Adoptive transfer of clone TCR containing T cells
	CAR-T cell therapy **	Adoptive transfer of genetically modified CAR-T cells
Other Personalized therapy	AlloVax	Personalized cancer vaccine from the patient’s tumor
	PNeoVCA	Personalized peptide-based vaccine with 20 neoantigens
	PANDA-VAC	Personalized and adjusted neoantigen peptide vaccine

ADCC—Antigen-dependent cell cytotoxicity; CAR—Chimeric antigen receptor; CD—Cluster of differentiation; CTLs—Cytolytic T lymphocytes; EGFr—Epidermal growth factor receptor; HPV—Human papilloma virus; hTERT—human telomerase reverse transcriptase; NK—Natural Killer; PI3K—Phosphoinositide 3-kinases; TCR—T cell receptor. * Given Fast Track status by the FDA; ** approved by the FDA for clinical use in cancer. References [3,4,132,161,177,178,179].

## Data Availability

Not applicable.

## References

[1] Waldman A.D., Fritz J.M., Lenardo M.J. (2020). A Guide to Cancer Immunotherapy: From T Cell Basic Science to Clinical Practice. Nat. Rev. Immunol..

[2] Blass E., Ott P.A. (2021). Advances in the Development of Personalized Neoantigen-Based Therapeutic Cancer Vaccines. Nat. Rev. Clin. Oncol..

[3] Devaraja K. (2019). Current Prospects of Molecular Therapeutics in Head and Neck Squamous Cell Carcinoma. Pharm. Med..

[4] Devaraja K., Aggarwal S., Singh M. (2023). Therapeutic Vaccination in Head and Neck Squamous Cell Carcinoma—A Review. Vaccines.

[5] Levine D.B. (2008). The Hospital for the Ruptured and Crippled: William Bradley Coley, Third Surgeon-in-Chief 1925–1933. HSS J..

[6] Hobohm U. (2001). Fever and Cancer in Perspective. Cancer Immunol. Immunother..

[7] Oiseth S.J., Aziz M.S. (2017). Cancer Immunotherapy: A Brief Review of the History, Possibilities, and Challenges Ahead. J. Cancer Metastasis Treat..

[8] Suzuki A., Abe S., Koyama K., Suzuki S., Nagao M., Kobayashi M., Nomura J., Tsutsumi T., Takeda T., Oka Y. (2021). Spontaneous Regression of Blastic Plasmacytoid Dendritic Cell Neoplasm Following Sepsis by Serratia Marcescens: A Case Report and Literature Review. Intern. Med..

[9] Burnet M. (1957). Cancer; a Biological Approach. I. The Processes of Control. Br. Med. J..

[10] Thomas L., Lawrence H. (1959). Cellular and Humoral Aspects of the Hypersensitive States.

[11] McCarthy E.F. (2006). The Toxins of William B. Coley and the Treatment of Bone and Soft-Tissue Sarcomas. Iowa Orthop. J..

[12] Busch W. (1868). Aus Der Sitzung Der Medicinischen Section Vom 13 November 1867. Berl. Klin. Wochenschr..

[13] Fehleisen N. (1882). Ueber die Züchtung der Erysipelkokken auf künstlichem Nährboden und ihre Uebertragbarkeit auf den Menschen. Dtsch. Med. Wochenschr..

[14] Bruns P. (1887). von Die Heilwirkung Des Erysipels Auf Geschwulste. Beitr. Klin. Chir..

[15] Coley W.B. (1910). The Treatment of Inoperable Sarcoma by Bacterial Toxins (The Mixed Toxins of the *Streptococcus erysipelas* and the *Bacillus prodigiosus*). Proc. R. Soc. Med..

[16] Coley W.B. (1891). Contribution to the Knowledge of Sarcoma. Ann. Surg..

[17] Ehrlich P. (1909). Ueber Den Jetzigen Stand Der Karzinomforschung. Weekblad Jaargang Eerst Helft.

[18] Miller J.F. (1961). Immunological Function of the Thymus. Lancet.

[19] Miller J.F., Mitchell G.F., Weiss N.S. (1967). Cellular Basis of the Immunological Defects in Thymectomized Mice. Nature.

[20] Burnet M. (1962). Role of the Thymus and Related Organs in Immunity. Br. Med. J..

[21] Claman H.N., Chaperon E.A., Triplett R.F. (1966). Thymus-Marrow Cell Combinations. Synergism in Antibody Production. Proc. Soc. Exp. Biol. Med..

[22] Wagner H., Röllinghoff M., Nossal G.J. (1973). T-Cell-Mediated Immune Responses Induced in Vitro: A Probe for Allograft and Tumor Immunity. Transplant. Rev..

[23] Carlson R.D., Flickinger J.C., Snook A.E. (2020). Talkin’ Toxins: From Coley’s to Modern Cancer Immunotherapy. Toxins.

[24] Blevins S.M., Bronze M.S. (2010). Robert Koch and the “golden Age” of Bacteriology. Int. J. Infect. Dis..

[25] Loeffler F. (1884). Untersuchungen Über Die Bedeutung Der Mikroorganismen Für Die Entstehung Der Diphtherie Beim Menschen, Bei Der Taube Und Beim Kalbe.

[26] Winau F., Winau R. (2002). Emil von Behring and Serum Therapy. Microbes Infect..

[27] Emil von B. Über Die Ursache Der Immunität von Ratten Gegen Milzbrand.: Centralblatt für Klinische Medicin 38. 1888; pp. 681–690.

[28] Von Behring E., Kitasato S. (1890). Uber Das Zustandekommen Der Diphtherie-Immunitat und Der Tetanus-Immunitat Bei Thieren. Deut. Med. Wochenschr..

[29] Winau F., Westphal O., Winau R. (2004). Paul Ehrlich—In Search of the Magic Bullet. Microbes Infect..

[30] Ehrlich P. (1901). Die Seitenkettentheorie Und Ihre Gegner. Münchner. Med. Wochenschr..

[31] Currie G.A. (1972). Eighty Years of Immunotherapy: A Review of Immunological Methods Used for the Treatment of Human Cancer. Br. J. Cancer.

[32] Von Leyden V., Blumenthal F. (1902). Vorläufige Mittheilungen Über Einige Ergebnisse Der Krebsforshung Auf Der 1. Medizinschen Klinik. Dtsch. Med. Wschr..

[33] Risley E.H. (1911). The Gilman–Coca Vaccine Emulsion Treatment of Cancer. Boston Med. Surg. J..

[34] Vaughan J.W. (1914). Cancer Vaccine and Anticancer Globulins as an Aid in the Surgical Treatment of Malignancy. J. Am. Med. Assoc..

[35] Dobosz P., Dzieciątkowski T. (2019). The Intriguing History of Cancer Immunotherapy. Front. Immunol..

[36] Dock G. (1904). The Influence of Complicating Diseases upon Leukaemia. Am. J. Med. Sci. (1827–1924).

[37] Larson C., Oronsky B., Scicinski J., Fanger G.R., Stirn M., Oronsky A., Reid T.R. (2015). Going Viral: A Review of Replication-Selective Oncolytic Adenoviruses. Oncotarget.

[38] Jensen C. (1903). Experimental Studies on Cancer in Mice (from German). Zentralblatt Bacteriol. Parasitenkd. Infekt..

[39] Little C.C. (1914). A possible mendelian explanation for a type of inheritance apparently non-mendelian in nature. Science.

[40] Murphy J.B., Morton J.J. (1915). The Lymphocyte as a Factor in Natural and Induced Resistance to Transplanted Cancer. Proc. Natl. Acad. Sci. USA.

[41] The CRI Timeline. https://www.cancerresearch.org/timeline.

[42] Everson T.C., Cole W.H. (1959). Spontaneous Regression of Malignant Disease. J. Am. Med. Assoc..

[43] Old L.J., Clarke D.A., Benacerraf B. (1959). Effect of Bacillus Calmette-Guerin Infection on Transplanted Tumours in the Mouse. Nature.

[44] Silverstein A.M. (2016). The Curious Case of the 1960 Nobel Prize to Burnet and Medawar. Immunology.

[45] Liston A. (2011). Immunological Tolerance 50 Years after the Burnet Nobel Prize. Immunol. Cell Biol..

[46] Gowans J.L., McGregor D.D., Cowen D.M. (1962). Initiation of Immune Responses by Small Lymphocytes. Nature.

[47] Miller J.F., Grant G.A., Roe F.J. (1963). Effect of thymectomy on the induction of skin tumours by 3,4-benzopyrene. Nature.

[48] Miller J.F. (1964). Influence of thymectomy on tumor induction by polyoma virus in C57BL mice. Proc. Soc. Exp. Biol. Med..

[49] Miller J.F. (2004). Events That Led to the Discovery of T-Cell Development and Function—A Personal Recollection. Tissue Antigens.

[50] Miller J. (2020). The Early Work on the Discovery of the Function of the Thymus, an Interview with Jacques Miller. Cell Death Differ..

[51] Cerottini J.C., Nordin A.A., Brunner K.T. (1970). In Vitro Cytotoxic Activity of Thymus Cells Sensitized to Alloantigens. Nature.

[52] Cerottini J.C., Nordin A.A., Brunner K.T. (1970). Specific in Vitro Cytotoxicity of Thymus-Derived Lymphocytes Sensitized to Alloantigens. Nature.

[53] Steinman R.M., Cohn Z.A. (1973). Identification of a Novel Cell Type in Peripheral Lymphoid Organs of Mice. I. Morphology, Quantitation, Tissue Distribution. J. Exp. Med..

[54] Banchereau J., Steinman R.M. (1998). Dendritic Cells and the Control of Immunity. Nature.

[55] Geissmann F., Gordon S., Hume D.A., Mowat A.M., Randolph G.J. (2010). Unravelling Mononuclear Phagocyte Heterogeneity. Nat. Rev. Immunol..

[56] Zinkernagel R.M., Doherty P.C. (1974). Restriction of in Vitro T Cell-Mediated Cytotoxicity in Lymphocytic Choriomeningitis within a Syngeneic or Semiallogeneic System. Nature.

[57] Zinkernagel R.M., Doherty P.C. (1974). Immunological Surveillance against Altered Self Components by Sensitised T Lymphocytes in Lymphocytic Choriomeningitis. Nature.

[58] Doherty P.C. (2007). Challenged by Complexity: My Twentieth Century in Immunology. Annu. Rev. Immunol..

[59] Raju T.N. (2000). The Nobel Chronicles. 1996: Peter Charles Doherty (b 1940) and Rolf M Zinkernagel (b 1944). Lancet.

[60] Volchenkov R., Sprater F., Vogelsang P., Appel S. (2012). The 2011 Nobel Prize in Physiology or Medicine. Scand. J. Immunol..

[61] Scott A.M., Allison J.P., Wolchok J.D. (2012). Monoclonal Antibodies in Cancer Therapy. Cancer Immun..

[62] Damián-Blanco P., Ahuexoteco-Sánchez S., Carbajal-Gallardo A.A., Coctecon-Chávelas F.C., Rodríguez-Nava C., Vences-Velázquez A., Medina-Flores Y., Mata-Ruíz O., Lloret-Sánchez L., Cortés-Sarabia K. (2023). Use of Monoclonal Antibodies in Cancer Immunotherapy: Types and Mechanisms of Action. Bol. Med. Hosp. Infant. Mex..

[63] Schwaber J., Cohen E.P. (1973). Human x Mouse Somatic Cell Hybrid Clone Secreting Immunoglobulins of Both Parental Types. Nature.

[64] Köhler G., Milstein C. (1975). Continuous Cultures of Fused Cells Secreting Antibody of Predefined Specificity. Nature.

[65] Ribatti D. (2014). From the Discovery of Monoclonal Antibodies to Their Therapeutic Application: An Historical Reappraisal. Immunol. Lett..

[66] Liu J.K.H. (2014). The History of Monoclonal Antibody Development—Progress, Remaining Challenges and Future Innovations. Ann. Med. Surg..

[67] Kaunitz J.D. (2017). Development of Monoclonal Antibodies: The Dawn of mAb Rule. Dig. Dis. Sci..

[68] Herberman R.B., Nunn M.E., Lavrin D.H., Asofsky R. (1973). Effect of Antibody to Theta Antigen on Cell-Mediated Immunity Induced in Syngeneic Mice by Murine Sarcoma Virus. J. Natl. Cancer Inst..

[69] Herberman R.B. (1973). In Vivo and in Vitro Assays of Cellular Immunity to Human Tumor Antigens. Fed. Proc..

[70] Kiessling R., Klein E., Wigzell H. (1975). “Natural” Killer Cells in the Mouse. I. Cytotoxic Cells with Specificity for Mouse Moloney Leukemia Cells. Specificity and Distribution According to Genotype. Eur. J. Immunol..

[71] Kiessling R., Klein E., Pross H., Wigzell H. (1975). “Natural” Killer Cells in the Mouse. II. Cytotoxic Cells with Specificity for Mouse Moloney Leukemia Cells. Characteristics of the Killer Cell. Eur. J. Immunol..

[72] Kiessling R., Petrányi G., Klein G., Wigzell H. (1976). Non-T-Cell Resistance against a Mouse Moloney Lymphoma. Int. J. Cancer.

[73] Chen J., Gao L., Wu X., Fan Y., Liu M., Peng L., Song J., Li B., Liu A., Bao F. (2023). BCG-Induced Trained Immunity: History, Mechanisms and Potential Applications. J. Transl. Med..

[74] Pearl R. (1928). On the Pathological Relations Between Cancer and Tuberculosis. Proc. Soc. Exp. Biol. Med..

[75] Morales A., Eidinger D. (1976). Bacillus Calmette-Guerin in the Treatment of Adenocarcinoma of the Kidney. J. Urol..

[76] Morales A., Eidinger D., Bruce A.W. (1976). Intracavitary Bacillus Calmette-Guerin in the Treatment of Superficial Bladder Tumors. J. Urol..

[77] Hanna M.G., Peters L.C. (1978). Specific Immunotherapy of Established Visceral Micrometastases by BCG-Tumor Cell Vaccine Alone or as an Adjunct to Surgery. Cancer.

[78] Grant R.M., Mackie R., Cochran A.J., Murray E.L., Hoyle D., Ross C. (1974). Results of Administering B.C.G. to Patients with Melanoma. Lancet.

[79] Donaldson R.C. (1973). Chemoimmunotherapy for Cancer of the Head and Neck. Am. J. Surg..

[80] Carswell E.A., Old L.J., Kassel R.L., Green S., Fiore N., Williamson B. (1975). An Endotoxin-Induced Serum Factor That Causes Necrosis of Tumors. Proc. Natl. Acad. Sci. USA.

[81] Old L.J. (1977). Cancer Immunology. Sci. Am..

[82] Ashley M.P., Zbar B., Hunter J.T., Rapp H.J., Sugimoto T. (1980). Adjuvant-Antigen Requirements for Active Specific Immunotherapy of Microscopic Metastases Remaining after Surgery. Cancer Res..

[83] Sukumar S., Hunter J.T., Terata N., Rapp H.J. (1983). Eradication of Microscopic Hepatic Metastases by Active Specific Immunization. Cancer Immunol. Immunother..

[84] Bier H., Armonat G., Bier J., Schirrmacher V., Ganzer U. (1989). Postoperative Active-Specific Immunotherapy of Lymph Node Micrometastasis in a Guinea Pig Tumor Model. Orl J. Otorhinolaryngol. Relat. Spec..

[85] Morton D.L., Foshag L.J., Hoon D.S., Nizze J.A., Famatiga E., Wanek L.A., Chang C., Davtyan D.G., Gupta R.K., Elashoff R. (1992). Prolongation of Survival in Metastatic Melanoma after Active Specific Immunotherapy with a New Polyvalent Melanoma Vaccine. Ann. Surg..

[86] Böhle A., Brandau S. (2003). Immune Mechanisms in Bacillus Calmette-Guerin Immunotherapy for Superficial Bladder Cancer. J. Urol..

[87] de Jong W.H., Teppema J.S., Wagenaar S.S., Paques M., Steerenberg P.A., Ruitenberg E.J. (1986). Histological Evaluation of Immunologically Mediated Tumor Regression of the Line 10 Guinea Pig Hepatocarcinoma. Virchows Arch. B Cell Pathol. Incl. Mol. Pathol..

[88] Miyazaki J., Onozawa M., Takaoka E., Yano I. (2018). Bacillus Calmette-Guérin Strain Differences as the Basis for Immunotherapies against Bladder Cancer. Int. J. Urol..

[89] Klein E. (1982). Lymphocyte-Mediated Lysis of Tumor Cells in Vitro. Antigen-Restricted Clonal and Unrestricted Polyclonal Effects. Springer Semin. Immunopathol..

[90] Stauss H.J., Van Waes C., Fink M.A., Starr B., Schreiber H. (1986). Identification of a Unique Tumor Antigen as Rejection Antigen by Molecular Cloning and Gene Transfer. J. Exp. Med..

[91] Cole W.H. (1981). Efforts to Explain Spontaneous Regression of Cancer. J. Surg. Oncol..

[92] Thomas L. (1982). On Immunosurveillance in Human Cancer. Yale J. Biol. Med..

[93] Burnet M. (1964). Donn. Br. Med. Bull..

[94] Ribatti D. (2017). The Concept of Immune Surveillance against Tumors. The First Theories. Oncotarget.

[95] Mitchell M.S., Kan-Mitchell J., Kempf R.A., Harel W., Shau H.Y., Lind S. (1988). Active Specific Immunotherapy for Melanoma: Phase I Trial of Allogeneic Lysates and a Novel Adjuvant. Cancer Res..

[96] Mitchell M.S., Harel W., Kempf R.A., Hu E., Kan-Mitchell J., Boswell W.D., Dean G., Stevenson L. (1990). Active-Specific Immunotherapy for Melanoma. J. Clin. Oncol..

[97] Mitchell M.S. (1998). Perspective on Allogeneic Melanoma Lysates in Active Specific Immunotherapy. Semin. Oncol..

[98] Sondak V.K., Sosman J.A. (2003). Results of Clinical Trials with an Allogenic Melanoma Tumor Cell Lysate Vaccine: Melacine. Semin. Cancer Biol..

[99] Sondak V.K., Liu P.-Y., Tuthill R.J., Kempf R.A., Unger J.M., Sosman J.A., Thompson J.A., Weiss G.R., Redman B.G., Jakowatz J.G. (2002). Adjuvant Immunotherapy of Resected, Intermediate-Thickness, Node-Negative Melanoma with an Allogeneic Tumor Vaccine: Overall Results of a Randomized Trial of the Southwest Oncology Group. J. Clin. Oncol..

[100] Morton D.L., Mozzillo N., Thompson J.F., Kelley M.C., Faries M., Wagner J., Schneebaum S., Schuchter L., Gammon G., Elashoff R. (2007). An International, Randomized, Phase III Trial of Bacillus Calmette-Guerin (BCG) plus Allogeneic Melanoma Vaccine (MCV) or Placebo after Complete Resection of Melanoma Metastatic to Regional or Distant Sites. JCO.

[101] Apostólico J.d.S., Lunardelli V.A.S., Coirada F.C., Boscardin S.B., Rosa D.S. (2016). Adjuvants: Classification, Modus Operandi, and Licensing. J. Immunol. Res..

[102] Firdaus F.Z., Skwarczynski M., Toth I. (2022). Developments in Vaccine Adjuvants. Methods Mol. Biol..

[103] Czerkinsky C.C., Nilsson L.A., Nygren H., Ouchterlony O., Tarkowski A. (1983). A Solid-Phase Enzyme-Linked Immunospot (ELISPOT) Assay for Enumeration of Specific Antibody-Secreting Cells. J. Immunol. Methods.

[104] Slota M., Lim J.-B., Dang Y., Disis M.L. (2011). ELISpot for Measuring Human Immune Responses to Vaccines. Expert. Rev. Vaccines.

[105] Ranieri E., Netti G.S., Gigante M. (2021). CTL ELISPOT Assay and T Cell Detection. Methods Mol. Biol..

[106] Saxena M., Bhardwaj N. (2017). Turbocharging Vaccines: Emerging Adjuvants for Dendritic Cell Based Therapeutic Cancer Vaccines. Curr. Opin. Immunol..

[107] Inaba K., Inaba M., Romani N., Aya H., Deguchi M., Ikehara S., Muramatsu S., Steinman R.M. (1992). Generation of Large Numbers of Dendritic Cells from Mouse Bone Marrow Cultures Supplemented with Granulocyte/Macrophage Colony-Stimulating Factor. J. Exp. Med..

[108] Young J.W., Szabolcs P., Moore M.A. (1995). Identification of Dendritic Cell Colony-Forming Units among Normal Human CD34+ Bone Marrow Progenitors That Are Expanded by c-Kit-Ligand and Yield Pure Dendritic Cell Colonies in the Presence of Granulocyte/Macrophage Colony-Stimulating Factor and Tumor Necrosis Factor Alpha. J. Exp. Med..

[109] De Plaen E., Lurquin C., Van Pel A., Mariamé B., Szikora J.P., Wölfel T., Sibille C., Chomez P., Boon T. (1988). Immunogenic (Tum-) Variants of Mouse Tumor P815: Cloning of the Gene of Tum- Antigen P91A and Identification of the Tum- Mutation. Proc. Natl. Acad. Sci. USA.

[110] van der Bruggen P., Traversari C., Chomez P., Lurquin C., De Plaen E., Van den Eynde B., Knuth A., Boon T. (1991). A Gene Encoding an Antigen Recognized by Cytolytic T Lymphocytes on a Human Melanoma. Science.

[111] Boon T., van der Bruggen P. (1996). Human Tumor Antigens Recognized by T Lymphocytes. J. Exp. Med..

[112] Wang R.F., Rosenberg S.A. (1999). Human Tumor Antigens for Cancer Vaccine Development. Immunol. Rev..

[113] Devaraja K., Aggarwal S., Verma S.S., Gupta S.C. (2020). Clinico-Pathological Peculiarities of Human Papilloma Virus Driven Head and Neck Squamous Cell Carcinoma: A Comprehensive Update. Life Sci..

[114] Coulie P.G., Van den Eynde B.J., van der Bruggen P., Boon T. (2014). Tumour Antigens Recognized by T Lymphocytes: At the Core of Cancer Immunotherapy. Nat. Rev. Cancer.

[115] Zarour H.M., DeLeo A., Finn O.J., Storkus W.J. (2003). Categories of Tumor Antigens. Holland-Frei Cancer Medicine.

[116] Kosaka A., Yajima Y., Hatayama M., Ikuta K., Sasaki T., Hirai N., Yasuda S., Nagata M., Hayashi R., Harabuchi S. (2021). A Stealth Antigen SPESP1, Which Is Epigenetically Silenced in Tumors, Is a Suitable Target for Cancer Immunotherapy. Cancer Sci..

[117] Yajima Y., Kosaka A., Ishibashi K., Yasuda S., Komatsuda H., Nagato T., Oikawa K., Kitada M., Takekawa M., Kumai T. (2022). A Tumor Metastasis-Associated Molecule TWIST1 Is a Favorable Target for Cancer Immunotherapy Due to Its Immunogenicity. Cancer Sci..

[118] Cheever M.A., Allison J.P., Ferris A.S., Finn O.J., Hastings B.M., Hecht T.T., Mellman I., Prindiville S.A., Viner J.L., Weiner L.M. (2009). The Prioritization of Cancer Antigens: A National Cancer Institute Pilot Project for the Acceleration of Translational Research. Clin. Cancer Res..

[119] Higano C.S., Schellhammer P.F., Small E.J., Burch P.A., Nemunaitis J., Yuh L., Provost N., Frohlich M.W. (2009). Integrated Data from 2 Randomized, Double-Blind, Placebo-Controlled, Phase 3 Trials of Active Cellular Immunotherapy with Sipuleucel-T in Advanced Prostate Cancer. Cancer.

[120] Burch P.A., Croghan G.A., Gastineau D.A., Jones L.A., Kaur J.S., Kylstra J.W., Richardson R.L., Valone F.H., Vuk-Pavlović S. (2004). Immunotherapy (APC8015, Provenge) Targeting Prostatic Acid Phosphatase Can Induce Durable Remission of Metastatic Androgen-Independent Prostate Cancer: A Phase 2 Trial. Prostate.

[121] Kantoff P.W., Higano C.S., Shore N.D., Berger E.R., Small E.J., Penson D.F., Redfern C.H., Ferrari A.C., Dreicer R., Sims R.B. (2010). Sipuleucel-T Immunotherapy for Castration-Resistant Prostate Cancer. N. Engl. J. Med..

[122] Cheever M.A., Higano C.S. (2011). PROVENGE (Sipuleucel-T) in Prostate Cancer: The First FDA-Approved Therapeutic Cancer Vaccine. Clin. Cancer Res..

[123] Brunet J.F., Denizot F., Luciani M.F., Roux-Dosseto M., Suzan M., Mattei M.G., Golstein P. (1987). A New Member of the Immunoglobulin Superfamily—CTLA-4. Nature.

[124] Linsley P.S., Wallace P.M., Johnson J., Gibson M.G., Greene J.L., Ledbetter J.A., Singh C., Tepper M.A. (1992). Immunosuppression in Vivo by a Soluble Form of the CTLA-4 T Cell Activation Molecule. Science.

[125] Iwai Y., Ishida M., Tanaka Y., Okazaki T., Honjo T., Minato N. (2002). Involvement of PD-L1 on Tumor Cells in the Escape from Host Immune System and Tumor Immunotherapy by PD-L1 Blockade. Proc. Natl. Acad. Sci. USA.

[126] Dong H., Strome S.E., Salomao D.R., Tamura H., Hirano F., Flies D.B., Roche P.C., Lu J., Zhu G., Tamada K. (2002). Tumor-Associated B7-H1 Promotes T-Cell Apoptosis: A Potential Mechanism of Immune Evasion. Nat. Med..

[127] Chen D.S., Mellman I. (2017). Elements of Cancer Immunity and the Cancer-Immune Set Point. Nature.

[128] Chen Y.-S., Shen C.-R. (2015). Immune Checkpoint Blockade Therapy: The 2014 Tang Prize in Biopharmaceutical Science. Biomed. J..

[129] Huang P.-W., Chang J.W.-C. (2019). Immune Checkpoint Inhibitors Win the 2018 Nobel Prize. Biomed. J..

[130] Dunn G.P., Bruce A.T., Ikeda H., Old L.J., Schreiber R.D. (2002). Cancer Immunoediting: From Immunosurveillance to Tumor Escape. Nat. Immunol..

[131] Dunn G.P., Old L.J., Schreiber R.D. (2004). The Immunobiology of Cancer Immunosurveillance and Immunoediting. Immunity.

[132] Song Q., Zhang C.-D., Wu X.-H. (2018). Therapeutic Cancer Vaccines: From Initial Findings to Prospects. Immunol. Lett..

[133] Shibata H., Xu N., Saito S., Zhou L., Ozgenc I., Webb J., Fu C., Zolkind P., Egloff A.M., Uppaluri R. (2021). Integrating CD4+ T Cell Help for Therapeutic Cancer Vaccination in a Preclinical Head and Neck Cancer Model. Oncoimmunology.

[134] Wei C., Ma Y., Wang F., Liao Y., Chen Y., Zhao B., Zhao Q., Wang D., Tang D. (2022). Igniting Hope for Tumor Immunotherapy: Promoting the “Hot and Cold” Tumor Transition. Clin. Med. Insights Oncol..

[135] Smyth M.J., Godfrey D.I., Trapani J.A. (2001). A Fresh Look at Tumor Immunosurveillance and Immunotherapy. Nat. Immunol..

[136] Hanahan D., Weinberg R.A. (2011). Hallmarks of Cancer: The next Generation. Cell.

[137] Hegde P.S., Karanikas V., Evers S. (2016). The Where, the When, and the How of Immune Monitoring for Cancer Immunotherapies in the Era of Checkpoint Inhibition. Clin. Cancer Res..

[138] de Visser K.E., Eichten A., Coussens L.M. (2006). Paradoxical Roles of the Immune System during Cancer Development. Nat. Rev. Cancer.

[139] Kather J.N., Suarez-Carmona M., Charoentong P., Weis C.-A., Hirsch D., Bankhead P., Horning M., Ferber D., Kel I., Herpel E. (2018). Topography of Cancer-Associated Immune Cells in Human Solid Tumors. eLife.

[140] Troiano G., Rubini C., Togni L., Caponio V.C.A., Zhurakivska K., Santarelli A., Cirillo N., Lo Muzio L., Mascitti M. (2020). The Immune Phenotype of Tongue Squamous Cell Carcinoma Predicts Early Relapse and Poor Prognosis. Cancer Med..

[141] Duan Q., Zhang H., Zheng J., Zhang L. (2020). Turning Cold into Hot: Firing up the Tumor Microenvironment. Trends Cancer.

[142] Rosenberg S.A., Spiess P., Lafreniere R. (1986). A New Approach to the Adoptive Immunotherapy of Cancer with Tumor-Infiltrating Lymphocytes. Science.

[143] Rosenberg S.A., Lotze M.T., Muul L.M., Chang A.E., Avis F.P., Leitman S., Linehan W.M., Robertson C.N., Lee R.E., Rubin J.T. (1987). A Progress Report on the Treatment of 157 Patients with Advanced Cancer Using Lymphokine-Activated Killer Cells and Interleukin-2 or High-Dose Interleukin-2 Alone. N. Engl. J. Med..

[144] Mitra A., Barua A., Huang L., Ganguly S., Feng Q., He B. (2023). From Bench to Bedside: The History and Progress of CAR T Cell Therapy. Front. Immunol..

[145] Pang Z., Wang Z., Li F., Feng C., Mu X. (2022). Current Progress of CAR-NK Therapy in Cancer Treatment. Cancers.

[146] Mellman I., Coukos G., Dranoff G. (2011). Cancer Immunotherapy Comes of Age. Nature.

[147] Kuwana Y., Asakura Y., Utsunomiya N., Nakanishi M., Arata Y., Itoh S., Nagase F., Kurosawa Y. (1987). Expression of Chimeric Receptor Composed of Immunoglobulin-Derived V Regions and T-Cell Receptor-Derived C Regions. Biochem. Biophys. Res. Commun..

[148] Gross G., Waks T., Eshhar Z. (1989). Expression of Immunoglobulin-T-Cell Receptor Chimeric Molecules as Functional Receptors with Antibody-Type Specificity. Proc. Natl. Acad. Sci. USA.

[149] Zhao J., Lin Q., Song Y., Liu D. (2018). Universal CARs, Universal T Cells, and Universal CAR T Cells. J. Hematol. Oncol..

[150] Demaria O., Gauthier L., Debroas G., Vivier E. (2021). Natural Killer Cell Engagers in Cancer Immunotherapy: Next Generation of Immuno-Oncology Treatments. Eur. J. Immunol..

[151] Tsao L.-C., Force J., Hartman Z.C. (2021). Mechanisms of Therapeutic Antitumor Monoclonal Antibodies. Cancer Res..

[152] Ramakrishnan M.S., Eswaraiah A., Crombet T., Piedra P., Saurez G., Iyer H., Arvind A.S. (2009). Nimotuzumab, a Promising Therapeutic Monoclonal for Treatment of Tumors of Epithelial Origin. MAbs.

[153] Parakh S., King D., Gan H.K., Scott A.M. (2020). Current Development of Monoclonal Antibodies in Cancer Therapy. Recent. Results Cancer Res..

[154] Thomas A., Teicher B.A., Hassan R. (2016). Antibody-Drug Conjugates for Cancer Therapy. Lancet Oncol..

[155] Akbari B., Farajnia S., Ahdi Khosroshahi S., Safari F., Yousefi M., Dariushnejad H., Rahbarnia L. (2017). Immunotoxins in Cancer Therapy: Review and Update. Int. Rev. Immunol..

[156] Waldhauer I., Steinle A. (2008). NK Cells and Cancer Immunosurveillance. Oncogene.

[157] Basílio-Queirós D., Mischak-Weissinger E. (2023). Natural Killer Cells- from Innate Cells to the Discovery of Adaptability. Front. Immunol..

[158] Nicholson S.E., Keating N., Belz G.T. (2019). Natural Killer Cells and Anti-Tumor Immunity. Mol. Immunol..

[159] Mizutani Y., Okada Y., Terachi T., Yoshida O. (1996). Prognostic Significance of Circulating Cytotoxic Lymphocytes against Autologous Tumors in Patients with Bladder Cancer. J. Urol..

[160] Imai K., Matsuyama S., Miyake S., Suga K., Nakachi K. (2000). Natural Cytotoxic Activity of Peripheral-Blood Lymphocytes and Cancer Incidence: An 11-Year Follow-up Study of a General Population. Lancet.

[161] Zhang M., Lam K.-P., Xu S. (2023). Natural Killer Cell Engagers (NKCEs): A New Frontier in Cancer Immunotherapy. Front. Immunol..

[162] Gauthier L., Morel A., Anceriz N., Rossi B., Blanchard-Alvarez A., Grondin G., Trichard S., Cesari C., Sapet M., Bosco F. (2019). Multifunctional Natural Killer Cell Engagers Targeting NKp46 Trigger Protective Tumor Immunity. Cell.

[163] Lee R.B., Maddineni S., Landry M., Diaz C., Tashfeen A., Yamada-Hunter S.A., Mackall C.L., Beinat C., Sunwoo J.B., Cochran J.R. (2024). An Engineered NKp46 Antibody for Construction of Multi-Specific NK Cell Engagers. Protein Eng. Des. Sel..

[164] Zhu A., Bai Y., Nan Y., Ju D. (2024). Natural Killer Cell Engagers: From Bi-Specific to Tri-Specific and Tetra-Specific Engagers for Enhanced Cancer Immunotherapy. Clin. Transl. Med..

[165] Sen R., Baltimore D. (1986). Multiple Nuclear Factors Interact with the Immunoglobulin Enhancer Sequences. Cell.

[166] Ghosh S., Hayden M.S. (2012). Celebrating 25 Years of NF-κB Research. Immunol. Rev..

[167] Bassères D.S., Baldwin A.S. (2006). Nuclear Factor-kappaB and Inhibitor of kappaB Kinase Pathways in Oncogenic Initiation and Progression. Oncogene.

[168] Zinatizadeh M.R., Schock B., Chalbatani G.M., Zarandi P.K., Jalali S.A., Miri S.R. (2021). The Nuclear Factor Kappa B (NF-kB) Signaling in Cancer Development and Immune Diseases. Genes Dis..

[169] Zou J.-Y., Chen Q.-L., Luo X.-C., Damdinjav D., Abdelmohsen U.R., Li H.-Y., Battulga T., Chen H.-B., Wang Y.-Q., Zhang J.-Y. (2024). Natural Products Reverse Cancer Multidrug Resistance. Front. Pharmacol..

[170] Vlahopoulos S.A. (2017). Aberrant Control of NF-κB in Cancer Permits Transcriptional and Phenotypic Plasticity, to Curtail Dependence on Host Tissue: Molecular Mode. Cancer Biol. Med..

[171] Yu H., Lin L., Zhang Z., Zhang H., Hu H. (2020). Targeting NF-κB Pathway for the Therapy of Diseases: Mechanism and Clinical Study. Signal Transduct. Target. Ther..

[172] Baud V., Karin M. (2009). Is NF-kappaB a Good Target for Cancer Therapy? Hopes and Pitfalls. Nat. Rev. Drug Discov..

[173] Li Y., Zhao B., Peng J., Tang H., Wang S., Peng S., Ye F., Wang J., Ouyang K., Li J. (2024). Inhibition of NF-κB Signaling Unveils Novel Strategies to Overcome Drug Resistance in Cancers. Drug Resist. Updat..

[174] Betzler A.C., Theodoraki M.-N., Schuler P.J., Döscher J., Laban S., Hoffmann T.K., Brunner C. (2020). NF-κB and Its Role in Checkpoint Control. Int. J. Mol. Sci..

[175] Grinberg-Bleyer Y., Oh H., Desrichard A., Bhatt D.M., Caron R., Chan T.A., Schmid R.M., Klein U., Hayden M.S., Ghosh S. (2017). NF-κB c-Rel Is Crucial for the Regulatory T Cell Immune Checkpoint in Cancer. Cell.

[176] Xia L., Tan S., Zhou Y., Lin J., Wang H., Oyang L., Tian Y., Liu L., Su M., Wang H. (2018). Role of the NFκB-Signaling Pathway in Cancer. Onco Targets Ther..

[177] Baldo B.A. (2016). Monoclonal Antibodies Approved for Cancer Therapy. Saf. Biol. Ther..

[178] Kumar M., Jalota A., Sahu S.K., Haque S. (2024). Therapeutic Antibodies for the Prevention and Treatment of Cancer. J. Biomed. Sci..

[179] Shiravand Y., Khodadadi F., Kashani S.M.A., Hosseini-Fard S.R., Hosseini S., Sadeghirad H., Ladwa R., O’Byrne K., Kulasinghe A. (2022). Immune Checkpoint Inhibitors in Cancer Therapy. Curr. Oncol..

[180] Wada S., Kobayashi S., Tsunoda T. (2022). Future Prospects for Cancer Immunotherapy—Strategies for Ineffective Cancers. Hum. Vaccines Immunother..

[181] Schaft N., Dörrie J., Schuler G., Schuler-Thurner B., Sallam H., Klein S., Eisenberg G., Frankenburg S., Lotem M., Khatib A. (2023). The Future of Affordable Cancer Immunotherapy. Front. Immunol..

